# Supplementary material for: Tropical cyclone exposure is associated with increased hospitalization rates in older adults
Source: Nat Commun. 2021 Mar 9;12:1545. doi: 10.1038/s41467-021-21777-1 (PMC7943804; doi:10.1038/s41467-021-21777-1)
Supplement: Supplementary file 1 — Supplementary Information [file 41467_2021_21777_MOESM1_ESM.pdf]

Supplementary Information

Tropical cyclone exposure is associated with increased hospitalization rates in older adults

Robbie M Parks, G Brooke Anderson, Rachel C Nethery, Ana Navas-Acien, Francesca Dominici, Marianthi-Anna Kioumourtzoglou

Supplementary Table 1. Number of hospitalizations and county-days, on days with and without exposure to tropical cyclones, over the study period (1999-2014) for causes included in the analysis.

Supplementary Table 2. ICD-9-CM codes per CCS Levels 1 and 3 used in analyses. ICD codes that were not included in the secondary CCS Level 3 analyses are grayed out.

Supplementary Table 1. Number of hospitalizations and county-days, on days with and without exposure to tropical cyclones, over the study period (1999-2014) for causes included in the analysis.

		**Number of hospitalizations**			**Number of county-days on which hospitalizations occurred**		**Mean Hospitalizations per county-day**	
			**Extreme wind events**					
		**Overall**	**Non-event**	**Event**	**Non-event**	**Event**	**Non-event**	**Event**
**Cause**	**Total**	69,682,674	69,372,155	310,519	47,508,591	207,120	1.46	1.50
	Cardiovascular diseases	20,927,807	20,834,060	93,747	12,524,689	54,248	1.66	1.73
	Respiratory diseases	8,710,722	8,675,609	35,113	5,449,502	21,758	1.59	1.61
	Cancers	3,903,821	3,886,744	17,077	3,410,016	14,893	1.14	1.15
	Injuries	6,234,960	6,204,465	30,495	4,603,906	21,558	1.35	1.41
	Neuropsychiatric disorders	1,651,738	1,644,437	7,301	1,341,122	5,845	1.23	1.25
	Blood diseases	887,242	883,344	3,898	736,453	3,210	1.20	1.21
	Digestive system diseases	7,133,839	7,102,395	31,444	5,679,962	24,862	1.25	1.26
	Endocrine disorders	2,802,636	2,790,028	12,608	1,981,817	8,749	1.41	1.44
	Genitourinary diseases	4,202,995	4,183,433	19,562	2,953,287	13,251	1.42	1.48
	Infectious and parasitic diseases	2,639,067	2,627,086	11,981	1,466,270	6,426	1.79	1.86
	Musculoskeletal and connective tissue diseases	4,303,980	4,285,867	18,113	2,779,233	11,509	1.54	1.57
	Nervous system diseases	1,485,787	1,478,922	6,865	1,294,839	5,903	1.14	1.16
	Skin and subcutaneous tissue diseases	1,156,623	1,150,960	5,663	854,199	4,065	1.35	1.39
	Other	3,641,457	3,624,805	16,652	2,433,296	10,843	1.49	1.54

Supplementary Table 2. ICD-9-CM codes per CCS Levels 1 and 3 used in analyses. ICD codes that were not included in the secondary CCS Level 3 analyses are grayed out.

CCS Level 1	CCS Level 1 description	CCS Level 3	CCS Level 3 description	ICD9-CM codes
1	Infectious and parasitic diseases	1	Tuberculosis	01000 01001 01002 01003 01004 01005 01006 01010 01011 01012 01013 01014 01015 01016 01080 01081 01082 01083 01084 01085 01086 01090 01091 01092 01093 01094 01095 01096 01100 01101 01102 01103 01104 01105 01106 01110 01111 01112 01113 01114 01115 01116 01120 01121 01122 01123 01124 01125 01126 01130 01131 01132 01133 01134 01135 01136 01140 01141 01142 01143 01144 01145 01146 01150 01151 01152 01153 01154 01155 01156 01160 01161 01162 01163 01164 01165 01166 01170 01171 01172 01173 01174 01175 01176 01180 01181 01182 01183 01184 01185 01186 01190 01191 01192 01193 01194 01195 01196 01200 01201 01202 01203 01204 01205 01206 01210 01211 01212 01213 01214 01215 01216 01220 01221 01222 01223 01224 01225 01226 01230 01231 01232 01233 01234 01235 01236 01280 01281 01282 01283 01284 01285 01286 01300 01301 01302 01303 01304 01305 01306 01310 01311 01312 01313 01314 01315 01316 01320 01321 01322 01323 01324 01325 01326 01330 01331 01332 01333 01334 01335 01336 01340 01341 01342 01343 01344 01345 01346 01350 01351 01352 01353 01354 01355 01356 01360 01361 01362 01363 01364 01365 01366 01380 01381 01382 01383 01384 01385 01386 01390 01391 01392 01393 01394 01395 01396 01400 01401 01402 01403 01404 01405 01406 01480 01481 01482 01483 01484 01485 01486 01500 01501 01502 01503 01504 01505 01506 01510 01511 01512 01513 01514 01515 01516 01520 01521 01522 01523 01524 01525 01526 01550 01551 01552 01553 01554 01555 01556 01560 01561 01562 01563 01564 01565 01566 01570 01571 01572 01573 01574 01575 01576 01580 01581 01582 01583 01584 01585 01586 01590 01591 01592 01593 01594 01595 01596 01600 01601 01602 01603 01604 01605 01606 01610 01611 01612 01613 01614 01615 01616 01620 01621 01622 01623 01624 01625 01626 01630 01631 01632 01633 01634 01635 01636 01640 01641 01642 01643 01644 01645 01646 01650 01651 01652 01653 01654 01655 01656 01660 01661 01662 01663 01664 01665 01666 01670 01671 01672 01673 01674 01676 01690 01691 01692 01693 01694 01695 01696 01700 01701 01702 01703 01704 01705 01706 01710 01711 01712 01713 01714 01715 01716 01720 01721 01722 01723 01724 01725 01726 01730 01731 01732 01733 01734 01735 01736 01740 01741 01742 01743 01744 01745 01746 01750 01751 01752 01753 01754 01755 01756 01760 01761 01762 01763 01764 01765 01766 01770 01771 01772 01773 01774 01775 01776 01780 01781 01782 01783 01784 01785 01786 01790 01791 01792 01793 01794 01795 01796 01800 01801 01802 01803 01804 01805 01806

				01880 01881 01882 01883 01884 01885 01886 01890 01891 01892 01893 01894 01895 01896 1370 1371 1372 1373 1374 V1201
		2	Septicemia except in labor	0031 0202 0223 0362 0380 0381 03810 03811 03812 03819 0382 0383 03840 03841 03842 03843 03844 03849 0388 0389 0545 449 77181 7907 99591 99592
		3	Bacterial infection (unspecified)	0200 0208 0209 0218 0219 0228 0229 0230 0231 0232 0233 0238 0239 024 025 0260 0269 0270 0271 0272 0278 0279 0300 0301 0302 0303 0308 0309 0312 0318 0319 03289 0329 0330 0331 0338 0339 0341 0363 03681 03689 0369 037 0392 0393 0394 0398 0399 0400 0401 0402 0403 04042 04081 04082 04089 0410 04100 04101 04102 04103 04104 04105 04109 0411 04110 04111 04112 04119 0412 0413 0414 04141 04142 04143 04149 0415 0416 0417 0418 04181 04182 04183 04184

				04185 04186 04189 0419 390 3929 7953 79531 79539 V090 V091 V092 V093 V094 V0950 V0951 V096 V0970 V0971 V0980 V0981 V0990 V0991
		4	Mycoses	1100 1101 1102 1103 1104 1105 1106 1108 1109 1110 1111 1112 1113 1118 1119 1120 1121 1122 1123 1125 11282 11284 11285 11289 1129 1141 1143 1149 11500 11509 11510 11519 11590 11599 1160 1161 1162 1170 1171 1172 1173 1174 1175 1176 1177 1178 1179 118
		5	HIV infection	042 0420 0421 0422 0429 0430 0431 0432 0433 0439 0440 0449 07953 27910 27919 79571 7958 V08
		6	Hepatitis	0700 0701 0702 07020 07021 07022 07023 0703 07030 07031 07032 07033 0704 07041 07042 07043 07044 07049 0705 07051 07052 07053 07054 07059 0706 07070 07071 0709 57140 57141 57142 57149 5731 5732 5733
		7	Viral infection	0500 0501 0502 0509 0510 05101 05102 0511 0512 0519 0522 0527 0528 0529 05310 05311 05312 05313 05314 05319 05371 05379 0538 0539 0540 05410 05411 05412 05413 05419 0542 0546 05471 05473 05474 05479 0548 0549 05579 0558 0559 05600 05609 05679 0568 0569 0570 0578 0579 05810 05811 05812 05881 05882 05889 05900 05901 05909 05910 05911 05912 05919 05920 05921 05922 0598 0599 0600 0601 0609 061 0650 0651 0652 0653 0654 0658 0659 0660 0661 0663 0664 06640 06641 06642 06649 0668 0669 071 0720 0723 07271 07272 07279 0728 0729 0737 0738 0739 0740 0741 0743 0748 075 0780 0781 07810 07811 07812 07819 0782 0783 0784 0785 0786 0787 07881 07882 07888 07889 0790 0791 0792 0793 0794 07950 07951 07952 07959 0796 0798 07981 07982 07983 07988 07989 0799 07998 07999 7908
		8	Other infections	080 0810 0811 0812 0819 0820 0821 0822 0823 08240 08241 08249 0828 0829 0831 0832 0838 0839 0840 0841 0842 0843 0844 0845 0846 0847 0848 0849 0850 0851 0852 0853 0854 0855 0859 0860 0861 0862 0863 0864 0865 0869 0870 0871 0879 0880 0888 08881 08882 08889 0889 1000 10089 1009 101 1020 1021 1022 1023 1024 1026 1027 1028 1029 1030 1031 1032 1033 1039 1040 1048 1049 1200 1201 1202 1203 1208 1209 1210 1212 1213 1214 1215 1216 1218 1219 1220 1221 1222 1223 1224 1225 1226 1227 1228 1229 1230 1231 1232 1233 1234 1235 1236 1238 1239 124 1250 1251 1252 1253 1254 1255 1256 1257 1259 1260 1261 1262 1263 1268 1269 1270 1271 1272 1273 1274 1275 1276 1277 1278 1279 1280 1281 1288 1289 129 1305 1307 1308 1309 13100 13101 13102 13103 13109 1318 1319 1320 1321 1322 1323 1329 1330 1338 1339 1340 1341 1342 1349 135 1360 1361 1362 13621 13629 1364 1365 1368 1369 1398 V120 V1200 V1203 V1204 V1209

		9	Sexually transmitted infections (not HIV or hepatitis)	0900 0901 0902 0903 09040 09041 09042 09049 0905 0906 0907 0909 0910 0911 0912 0913 0914 09150 09151 09152 09161 09162 09169 0917 09181 09182 09189 0919 0920 0929 0930 0931 09320 09321 09322 09323 09324 09381 09382 09389 0939 0940 0941 0942 0943 09481 09482 09483 09484 09485 09486 09487 09489 0949 0950 0951 0952 0953 0954 0955 0956 0957 0958 0959 096 0970 0971 0979 0980 09810 09811 09812 09813 09814 09815 09816 09817 09819 0982 09830 09831 09832 09833 09834 09835 09836 09837 09839 09840 09841 09842 09843 09849 09850 09851 09852 09853 09859 0986 0987 09881 09882 09883 09884 09885 09886 09889 0990 0991 0992 0993 0994 09940 09941 09949 09950 09951 09952 09953 09954 09955 09956 09959 0998 0999 79505 79515 79519 79675 79679
		10	Immunizations and screening	7955 79551 79552 7956 V010 V011 V012 V013 V014 V015 V016 V017 V0171 V0179 V018 V0181 V0182 V0183 V0184 V0189 V019 V020 V021 V022 V023 V024 V025 V0251 V0252 V0253 V0254 V0259 V026 V0260 V0261 V0262 V0269 V027 V028 V029 V030 V031 V032 V033 V034 V035 V036 V037 V038 V0381 V0382 V0389 V039 V040 V041 V042 V043 V044 V045 V046 V047 V048 V0481 V0482 V0489 V050 V051 V052 V053 V054 V058 V059 V060 V061 V062 V063 V064 V065 V066 V068 V069 V286 V712 V7182 V7183 V730 V731 V732 V733 V734 V735 V736 V738 V7381 V7388 V7389 V739 V7398 V7399 V740 V741 V742 V743 V744 V745 V746 V748 V749 V750 V751 V752 V753 V754 V755 V756 V757 V758 V759
2	Cancers	11	Head and neck	1400 1401 1403 1404 1405 1406 1408 1409 1410 1411 1412 1413 1414 1415 1416 1418 1419 1420 1421 1422 1428 1429 1430 1431 1438 1439 1440 1441 1448 1449 1450 1451 1452 1453 1454 1455 1456 1458 1459 1460 1461 1462 1463 1464 1465 1466 1467 1468 1469 1470 1471 1472 1473 1478 1479 1480 1481 1482 1483 1488 1489 1490 1491 1498 1499 1600 1601 1602 1603 1604 1605 1608 1609 1610 1611 1612 1613 1618 1619 1950 2300 2310 V1001 V1002 V1021
		12	Esophagus	1500 1501 1502 1503 1504 1505 1508 1509 2301 V1003
		13	Stomach	1510 1511 1512 1513 1514 1515 1516 1518 1519 20923 2302 V1004
		14	Colon	1530 1531 1532 1533 1534 1535 1536 1537 1538 1539 1590 20910 20911 20912 20913 20914 20915 20916 2303 V1005
		15	Rectum and anus	1540 1541 1542 1543 1548 20917 2304 2305 2306 79670 79671 79672 79673 79674 79676 V1006
		16	Liver and intrahepatic bile duct	1550 1551 1552 2308 V1007
		17	Pancreas	1570 1571 1572 1573 1574 1578 1579

		18	Gastrointestinal tract organs and perito-neum	1520 1521 1522 1523 1528 1529 1560 1561 1562 1568 1569 1580 1588 1589 1591 1598 1599 20900 20901 20902 20903 2307 2309 V1000 V1009
		19	Bronchus and lung	1622 1623 1624 1625 1628 1629 20921 2312 V1011
		20	Other respiratory and intrathoracic	1620 1630 1631 1638 1639 1650 1658 1659 2311 2318 2319 V1012 V1020 V1022
		21	Bone and connective tissue	1700 1701 1702 1703 1704 1705 1706 1707 1708 1709 1710 1712 1713 1714 1715 1716 1717 1718 1719
		22	Melanomas of skin	1720 1721 1722 1723 1724 1725 1726 1727 1728 1729 V1082
		23	Non-epithelial cancer of skin	1730 17300 17301 17302 17309 1731 17310 17311 17312 17319 1732 17320 17321 17322 17329 1733 17330 17331 17332 17339 1734 17340 17341 17342 17349 1735 17350 17351 17352 17359 1736 17360 17361 17362 17369 1737 17370 17371 17372 17379 1738 17380 17381 17382 17389 1739 17390 17391 17392 17399 20931 20932 20933 20934 20935 20936 2320 2321 2322 2323 2324 2325 2326 2327 2328 2329 V1083
		24	Breast	1740 1741 1742 1743 1744 1745 1746 1748 1749 1750 1759 2330 V103
		25	Uterus	179 1820 1821 1828 2332 V1042
		26	Cervix	1800 1801 1808 1809 2331 7950 79506 V1041
		27	Ovary	1830 V1043
		28	Other female genital organs	181 1832 1833 1834 1835 1838 1839 1840 1841 1842 1843 1844 1848 1849 2333 23330 23331 23332 23339 79516 V1040 V1044
		29	Prostate	185 2334 V1046
		30	Testis	1860 1869 V1047

		31	Other male genital organs	1871 1872 1873 1874 1875 1876 1877 1878 1879 2335 2336 V1045 V1048 V1049
		32	Bladder	1880 1881 1882 1883 1884 1885 1886 1887 1888 1889 2337 V1051
		33	Kidney and renal pelvis	1890 1891 20924 V1052 V1053
		34	Other urinary organs	1892 1893 1894 1898 1899 2339 V1050 V1059
		35	Brain and nervous system	1910 1911 1912 1913 1914 1915 1916 1917 1918 1919 1920 1921 1922 1923 1928 1929 V1085 V1086
		36	Thyroid	193 25802 25803 V1087
		37	Hodgkin's disease	20100 20101 20102 20103 20104 20105 20106 20107 20108 20110 20111 20112 20113 20114 20115 20116 20117 20118 20120 20121 20122 20123 20124 20125 20126 20127 20128 20140 20141 20142 20143 20144 20145 20146 20147 20148 20150 20151 20152 20153 20154 20155 20156 20157 20158 20160 20161 20162 20163 20164 20165 20166 20167 20168 20170 20171 20172 20173 20174 20175 20176 20177 20178 20190 20191 20192 20193 20194 20195 20196 20197 20198 V1072
		38	Non-Hodgkin's lymphoma	20000 20001 20002 20003 20004 20005 20006 20007 20008 20010 20011 20012 20013 20014 20015 20016 20017 20018 20020 20021 20022 20023 20024 20025 20026 20027 20028 20030 20031 20032 20033 20034 20035 20036 20037 20038 20040 20041 20042 20043 20044 20045 20046 20047 20048 20050 20051 20052 20053 20054 20055 20056 20057 20058 20060 20061 20062 20063 20064 20065 20066 20067 20068 20070 20071 20072 20073 20074 20075 20076 20077 20078 20080 20081 20082 20083 20084 20085 20086 20087 20088 20200 20201 20202 20203 20204 20205 20206 20207 20208 20210 20211 20212 20213 20214 20215 20216 20217 20218 20220 20221 20222 20223 20224 20225 20226 20227 20228 20270 20271 20272 20273 20274 20275 20276 20277 20278 20280 20281 20282 20283 20284 20285 20286 20287 20288 20290 20291 20292 20293 20294 20295 20296 20297 20298 V1071 V1079

		39	Leukemias	20240 20241 20242 20243 20244 20245 20246 20247 20248 2031 20310 20311 20312 2040 20400 20401 20402 2041 20410 20411 20412 2042 20420 20421 20422 2048 20480 20481 20482 2049 20490 20491 20492 2050 20500 20501 20502 2051 20510 20511 20512 2052 20520 20521 20522 2053 20530 20531 20532 2058 20580 20581 20582 2059 20590 20591 20592 2060 20600 20601 20602 2061 20610 20611 20612 2062 20620 20621 20622 2068 20680 20681 20682 2069 20690 20691 20692 2070 20700 20701 20702 2071 20710 20711 20712 2072 20720 20721 20722 2078 20780 20781 20782 2080 20800 20801 20802 2081 20810 20811 20812 2082 20820 20821 20822 2088 20880 20881 20882 2089 20890 20891 20892 V1060 V1061 V1062 V1063 V1069
		40	Multiple myeloma	2030 20300 20301 20302 2038 20380 20381 20382
		41	Other cancers	1640 1641 1642 1643 1648 1649 1760 1761 1762 1763 1764 1765 1768 1769 1900 1901 1902 1903 1904 1905 1906 1907 1908 1909 1940 1941 1943 1944 1945 1946 1948 1949 1951 1952 1953 1954 1955 1958 20230 20231 20232 20233 20234 20235 20236 20237 20238 20250 20251 20252 20253 20254 20255 20256 20257 20258 20260 20261 20262 20263 20264 20265 20266 20267 20268 20922 20925 20926 20927 2340 2348 2349 7951 79510 79511 79512 79513 79514 V1029 V1081 V1084 V1088 V1089 V109 V1090 V1091 V711
		42	Secondary malignancies	1960 1961 1962 1963 1965 1966 1968 1969 1970 1971 1972 1973 1974 1975 1976 1977 1978 1980 1981 1982 1983 1984 1985 1986 1987 19881 19882 19889 20971 20972 20973 20974 51181 78951
		43	Unspecified site	1990 1991 1992 20920 20929 20930 20970 20975 20979
		44	Cancers of unspecified nature or uncertain behavior	2350 2351 2352 2353 2354 2355 2356 2357 2358 2359 2360 2361 2362 2363 2364 2365 2366 2367 23690 23691 23699 2370 2371 2372 2373 2374 2375 2376 2377 23770 23771 23772 23773 23779 2379 2380 2381 2382 2383 2384 2385 2386 2387 23871 23872 23873 23874 23875 23876 23877 23879 2388 2389 2390 2391 2392 2393 2394 2395 2396 2397 2398 23981 23989 2399
		45	Chemotherapy and radiotherapy	V580 V581 V5811 V5812 V661 V662 V671 V672
		46	Uterus (benign)	2180 2181 2182 2189 2190 2191 2198 2199

		47	Other (benign)	20940 20941 20942 20943 20950 20951 20952 20953 20954 20955 20956 20957 20960 20961 20962 20963 20964 20965 20966 20967 20969 2100 2101 2102 2103 2104 2105 2106 2107 2108 2109 2110 2111 2112 2113 2114 2115 2116 2117 2118 2119 2120 2121 2122 2123 2124 2125 2126 2127 2128 2129 2130 2131 2132 2133 2134 2135 2136 2137 2138 2139 2140 2141 2142 2143 2144 2148 2149 2150 2152 2153 2154 2155 2156 2157 2158 2159 2160 2161 2162 2163 2164 2165 2166 2167 2168 2169 217 220 2210 2211 2212 2218 2219 2220 2221 2222 2223 2224 2228 2229 2230 2231 2232 2233 22381 22389 2239 2240 2241 2242 2243 2244 2245 2246 2247 2248 2249 2250 2251 2252 2253 2254 2258 2259 226 2270 2271 2273 2274 2275 2276 2278 2279 22800 22801 22802 22803 22804 22809 2281 2290 2298 2299 V1272
3	Endocrine disorders	48	Thyroid disorders	2400 2409 2410 2411 2419 24200 24201 24210 24211 24220 24221 24230 24231 24240 24241 24280 24281 24290 24291 243 2440 2441 2442 2443 2448 2449 2450 2451 2452 2453 2454 2458 2459 2460 2461 2462 2463 2468 2469 7945
		49	Diabetes mellitus without complication	24900 25000 25001 7902 79021 79022 79029 7915 7916 V4585 V5391 V6546
		50	Diabetes mellitus with complications	24901 24910 24911 24920 24921 24930 24931 24940 24941 24950 24951 24960 24961 24970 24971 24980 24981 24990 24991 25002 25003 25010 25011 25012 25013 25020 25021 25022 25023 25030 25031 25032 25033 25040 25041 25042 25043 25050 25051 25052 25053 25060 25061 25062 25063 25070 25071 25072 25073 25080 25081 25082 25083 25090 25091 25092 25093
		51	Other endocrine disorders	2510 2511 2512 2513 2514 2515 2518 2519 2520 25200 25201 25202 25208 2521 2528 2529 2530 2531 2532 2533 2534 2535 2536 2537 2538 2539 2540 2541 2548 2549 2550 2551 25510 25511 25512 25513 25514 2552 2553 2554 25541 25542 2555 2556 2558 2559 2560 2561 2562 2563 2564 2568 2569 2570 2571 2572 2578 2579 2580 25801 2581 2588 2589 2590 2591 2592 2593 2594 2595 25950 25951 25952 2598 2599 7946
		52	Nutritional deficiencies	260 261 262 2630 2631 2632 2638 2639 2640 2641 2642 2643 2644 2645 2646 2647 2648 2649 2650 2651 2652 2660 2661 2662 2669 267 2680 2681 2682 2689 2690 2691 2692 2693 2698 2699 7994 V121
		53	Disorders of lipid metabolism	2720 2721 2722 2723 2724

		54	Gout and other crystal arthropathies	2740 27400 27401 27402 27403 27410 27411 27419 27481 27482 27489 2749 71210 71211 71212 71213 71214 71215 71216 71217 71218 71219 71220 71221 71222 71223 71224 71225 71226 71227 71228 71229 71230 71231 71232 71233 71234 71235 71236 71237 71238 71239 71280 71281 71282 71283 71284 71285 71286 71287 71288 71289 71290 71291 71292 71293 71294 71295 71296 71297 71298 71299
		55	Fluid and electrolyte disorders	2760 2761 2762 2763 2764 2765 27650 27651 27652 2766 27669 2767 2768 2769 9951
		56	Cystic fibrosis	27700 27701 27702 27703 27709
		57	Immunity disorders	27900 27901 27902 27903 27904 27905 27906 27909 27911 27912 27913 2792 2793 2794 27941 27949 2798 2799
		58	Other disorders of stomach and duodenum	2700 2701 2702 2703 2704 2705 2706 2707 2708 2709 2710 2711 2712 2713 2714 2718 2719 2725 2726 2727 2728 2729 2730 2731 2732 2733 2734 2738 2739 2750 27501 27502 27503 27509 2751 2752 2753 2754 27540 27541 27542 27549 2755 2758 2759 2771 2772 2773 27730 27731 27739 2774 2775 2776 2777 2778 27781 27782 27784 27785 27786 27787 27789 2779 2780 27800 27801 27802 27803 2781 2782 2783 2784 2788 7831 7832 78321 78322 7833 7834 78340 78341 78342 78343 7835 7837 7839 79391 7947 7957 79579 V122 V1221 V1229 V850 V8521 V8522 V8523 V8524 V8525 V8530 V8531 V8532 V8533 V8534 V8535 V8536 V8537 V8538 V8539 V854 V8541 V8542 V8543 V8544 V8545 V8551 V8553 V8554
4	Blood diseases	59	Deficiency and other anemia	2800 2801 2808 2809 2810 2811 2812 2813 2814 2818 2819 2820 2821 2822 2823 2824 28240 28243 28244 28245 28246 28247 28249 2827 2828 2829 2830 2831 28310 28311 28319 2832 2839 2840 28401 28409 2841 28411 28412 28419 2842 2848 28481 28489 2849 2850 28521 28522 28529 2858 2859
		60	Acute posthemorrhagic anemia	2851
		61	Sickle cell anemia	28241 28242 2825 28260 28261 28262 28263 28264 28268 28269

		62	Coagulation and hemorrhagic disorders	2860 2861 2862 2863 2864 2865 28652 28653 28659 2866 2867 2869 2870 2871 2872 2873 28730 28731 28732 28733 28739 2874 28749 2875 2878 2879 28981 28982 28984 7827
		63	Diseases of white blood cells	2880 28800 28801 28802 28803 28804 28809 2881 2882 2883 2884 28850 28851 28859 28860 28861 28862 28863 28864 28865 28866 28869 2888 2889 28953
		64	Other hematologic conditions	2890 2894 28950 28951 28952 28959 2896 2897 2898 28983 28989 2899 7900 79001 79009 V123 V582
5	Neuropsychiatric disorders	650	Adjustment disorders	3090 3091 30922 30923 30924 30928 30929 3093 3094 30982 30983 30989 3099
		651	Anxiety disorders	29384 30000 30001 30002 30009 30010 30020 30021 30022 30023 30029 3003 3005 30089 3009 3080 3081 3082 3083 3084 3089 30981 3130 3131 31321 31322 3133 31382 31383
		652	Attention-deficit, conduct, and disruptive behavior disorders	31200 31201 31202 31203 31210 31211 31212 31213 31220 31221 31222 31223 3124 3128 31281 31282 31289 3129 31381 31400 31401 3141 3142 3148 3149
		653	Delirium and dementia	2900 29010 29011 29012 29013 29020 29021 2903 29040 29041 29042 29043 2908 2909 2930 2931 2940 2941 29410 29411 29420 29421 2948 2949 3100 3102 3108 31081 31089 3109 3310 3311 33111 33119 3312 33182 797
		654	Developmental disorders	3070 3079 31500 31501 31502 31509 3151 3152 31531 31532 31534 31535 31539 3154 3155 3158 3159 317 3180 3181 3182 319 V400 V401

		655	Disorders usually diagnosed in infancy, childhood, or adolescence	29900 29901 29910 29911 29980 29981 29990 29991 30720 30721 30722 30723 3073 3076 3077 30921 31323 31389 3139
		656	Impulse control disorders, NEC	31230 31231 31232 31233 31234 31235 31239
		657	Mood disorders	29383 29600 29601 29602 29603 29604 29605 29606 29610 29611 29612 29613 29614 29615 29616 29620 29621 29622 29623 29624 29625 29626 29630 29631 29632 29633 29634 29635 29636 29640 29641 29642 29643 29644 29645 29646 29650 29651 29652 29653 29654 29655 29656 29660 29661 29662 29663 29664 29665 29666 2967 29680 29681 29682 29689 29690 29699 3004 311
		658	Personality disorders	3010 30110 30111 30112 30113 30120 30121 30122 3013 3014 30150 30151 30159 3016 3017 30181 30182 30183 30184 30189 3019
		659	Schizophrenia and other psychotic disorders	29381 29382 29500 29501 29502 29503 29504 29505 29510 29511 29512 29513 29514 29515 29520 29521 29522 29523 29524 29525 29530 29531 29532 29533 29534 29535 29540 29541 29542 29543 29544 29545 29550 29551 29552 29553 29554 29555 29560 29561 29562 29563 29564 29565 29570 29571 29572 29573 29574 29575 29580 29581 29582 29583 29584 29585 29590 29591 29592 29593 29594 29595 2970 2971 2972 2973 2978 2979 2980 2981 2982 2983 2984 2988 2989
		660	Alcohol-related disorders	2910 2911 2912 2913 2914 2915 2918 29181 29182 29189 2919 30300 30301 30302 30303 30390 30391 30392 30393 30500 30501 30502 30503 76071 9800
		661	Substance-related disorders	2920 29211 29212 2922 29281 29282 29283 29284 29285 29289 2929 30400 30401 30402 30403 30410 30411 30412 30413 30420 30421 30422 30423 30430 30431 30432 30433 30440 30441 30442 30443 30450 30451 30452 30453 30460 30461 30462 30463 30470 30471 30472 30473 30480 30481 30482 30483 30490 30491 30492 30493 30520 30521 30522 30523 30530 30531 30532 30533 30540 30541 30542 30543 30550 30551 30552 30553 30560 30561 30562 30563 30570 30571 30572 30573 30580 30581 30582 30583 30590 30591 30592 30593 64830 64831 64832 64833 64834 65550 65551 65553 76072 76073 76075 7795 96500 96501 96502 96509 V6542

		662	Suicide and intentional self-inflicted injury	E9500 E9501 E9502 E9503 E9504 E9505 E9506 E9507 E9508 E9509 E9510 E9511 E9518 E9520 E9521 E9528 E9529 E9530 E9531 E9538 E9539 E954 E9550 E9551 E9552 E9553 E9554 E9555 E9556 E9557 E9559 E956 E9570 E9571 E9572 E9579 E9580 E9581 E9582 E9583 E9584 E9585 E9586 E9587 E9588 E9589 E959 V6284
		663	Screening and history of mental health and substance abuse codes	3051 30510 30511 30512 30513 33392 3575 4255 5353 53530 53531 5710 5711 5712 5713 7903 V110 V111 V112 V113 V114 V118 V119 V154 V1541 V1542 V1549 V1582 V6285 V663 V701 V702 V7101 V7102 V7109 V790 V791 V792 V793 V798 V799
		670	Miscellaneous mental health disorders	29389 2939 30011 30012 30013 30014 30015 30016 30019 3006 3007 30081 30082 3021 3022 3023 3024 30250 30251 30252 30253 3026 30270 30271 30272 30273 30274 30275 30276 30279 30281 30282 30283 30284 30285 30289 3029 3060 3061 3062 3063 3064 30650 30651 30652 30653 30659 3066 3067 3068 3069 3071 30740 30741 30742 30743 30744 30745 30746 30747 30748 30749 30750 30751 30752 30753 30754 30759 30780 30781 30789 3101 316 64840 64841 64842 64843 64844 V402 V403 V4031 V4039 V409 V673
6	Nervous system diseases	76	Meningitis	00321 0360 0470 0471 0478 0479 0490 0491 0530 05472 0721 10081 11283 1142 11501 11511 11591 3200 3201 3202 3203 3207 3208 32081 32082 32089 3209 3210 3211 3212 3213 3214 3218 3220 3221 3222 3229
		77	Encephalitis	0361 0462 0498 0499 0520 0543 0550 05601 05821 05829 0620 0621 0622 0623 0624 0625 0628 0629 0630 0631 0632 0638 0639 064 0662 0722 1300 1390 3230 32301 32302 3231 3232 3234 32341 32342 3235 32351 32352 3236 32361 32362 32363 3237 32371 32372 3238 32381 32382 3239 34120 34121 34122
		78	Other CNS infection and poliomyelitis	04500 04501 04502 04503 04510 04511 04512 04513 04520 04521 04522 04523 04590 04591 04592 04593 0460 0461 04611 04619 0463 04671 04672 04679 0468 0469 048 138 3240 3241 3249 326 V1202
		79	Parkinson's disease	3320
		80	Multiple sclerosis	340

		81	Other hereditary and degenerative nervous system conditions	3300 3301 3302 3303 3308 3309 3313 3314 3315 3316 3317 33181 33189 3319 3330 3331 3332 3333 3334 3335 3336 3337 33371 33372 33379 33381 33382 33383 33384 33385 33389 33390 33391 33393 33394 33399 3340 3341 3342 3343 3344 3348 3349 3350 33510 33511 33519 33520 33521 33522 33523 33524 33529 3358 3359 3360 3361 3362 3363 3368 3369 3370 33700 33701 33709 3371 3373 3379
		82	Paralysis	3420 34200 34201 34202 3421 34210 34211 34212 34280 34281 34282 3429 34290 34291 34292 3430 3431 3432 3433 3434 3438 3439 3440 34400 34401 34402 34403 34404 34409 3441 3442 3443 34430 34431 34432 3444 34440 34441 34442 3445 34460 3448 34481 34489 3449 78072 7814
		83	Epilepsy and convulsions	3450 34500 34501 3451 34510 34511 3452 3453 3454 34540 34541 3455 34550 34551 3456 34560 34561 3457 34570 34571 3458 34580 34581 3459 34590 34591 7803 78031 78032 78033 78039
		84	Headache (including migraine)	33900 33901 33902 33903 33904 33905 33909 33910 33911 33912 33920 33921 33922 3393 33941 33942 33943 33944 33981 33982 33983 33984 33985 33989 3460 34600 34601 34602 34603 3461 34610 34611 34612 34613 3462 34620 34621 34622 34623 34630 34631 34632 34633 34640 34641 34642 34643 34650 34651 34652 34653 34670 34671 34672 34673 3468 34680 34681 34682 34683 3469 34690 34691 34692 34693 7840
		85	Coma, stupor and brain damage	3481 7800 78001 78003 78009
		86	Cataract	36600 36601 36602 36603 36604 36609 36610 36611 36612 36613 36614 36615 36616 36617 36618 36619 36620 36621 36622 36623 36630 36631 36632 36633 36634 36641 36642 36643 36644 36645 36646 36650 36651 36652 36653 3668 3669 V431
		87	Retinal detachments, defects, vascular occlusion and retinopathy	36100 36101 36102 36103 36104 36105 36106 36107 36110 36111 36112 36113 36114 36119 3612 36130 36131 36132 36133 36181 36189 3619 36201 36202 36203 36204 36205 36206 36207 36210 36211 36212 36213 36214 36215 36216 36217 36218 36220 36221 36222 36223 36224 36225 36226 36227 36229 36230 36231 36232 36233 36234 36235 36236 36237 36240 36241 36242 36243 36250 36251 36252 36253 36254 36255 36256 36257 36260 36261 36262 36263 36264 36265 36266 36270 36271 36272 36273 36274 36275 36276 36277 36281 36282 36283 36284 36285 36289 3629

		88	Glaucoma	36500 36501 36502 36503 36504 36505 36506 36510 36511 36512 36513 36514 36515 36520 36521 36522 36523 36524 36531 36532 36541 36542 36543 36544 36551 36552 36559 36560 36561 36562 36563 36564 36565 36570 36571 36572 36573 36574 36581 36582 36583 36589 3659
		89	Blindness and vision defects	3670 3671 36720 36721 36722 36731 36732 3674 36751 36752 36753 36781 36789 3679 36800 36801 36802 36803 36810 36811 36812 36813 36814 36815 36816 3682 36830 36831 36832 36833 36834 36840 36841 36842 36843 36844 36845 36846 36847 36851 36852 36853 36854 36855 36859 36860 36861 36862 36863 36869 3688 3689 36900 36901 36902 36903 36904 36905 36906 36907 36908 36910 36911 36912 36913 36914 36915 36916 36917 36918 36920 36921 36922 36923 36924 36925 3693 3694 36960 36961 36962 36963 36964 36965 36966 36967 36968 36969 36970 36971 36972 36973 36974 36975 36976 3698 3699 V410
		90	Inflammation and infection of eye	0213 03281 05320 05321 05322 05329 05440 05441 05442 05443 05444 05449 05571 0760 0761 0769 0770 0771 0772 0773 0774 0778 0779 07798 07799 11502 11512 11592 1301 1302 1391 36000 36001 36002 36003 36004 36011 36012 36013 36014 36019 36300 36301 36303 36304 36305 36306 36307 36308 36310 36311 36312 36313 36314 36315 36320 36321 36322 36400 36401 36402 36403 36404 36405 36410 36411 36421 36422 36423 36424 3643 37020 37021 37022 37023 37024 37031 37032 37033 37034 37035 37040 37044 37049 37050 37052 37054 37055 37059 3708 3709 37200 37201 37202 37203 37204 37205 37206 37210 37211 37212 37213 37214 37215 37220 37221 37222 37230 37231 37233 37239 37300 37301 37302 37311 37312 37313 37331 37332 37333 37334 3734 3735 3736 3738 3739 37500 37501 37502 37503 37530 37531 37532 37533 37541 37542 37543 37600 37601 37602 37603 37604 37610 37611 37612 37613 37730 37731 37732 37733 37734 37739 37900 37901 37902 37903 37904 37905 37906 37907 37909 37960 37961 37962 37963

		91	Other eye disorders	36020 36021 36023 36024 36029 36030 36031 36032 36033 36034 36040 36041 36042 36043 36044 36050 36051 36052 36053 36054 36055 36059 36060 36061 36062 36063 36064 36065 36069 36081 36089 3609 36330 36331 36332 36333 36334 36335 36340 36341 36342 36343 36350 36351 36352 36353 36354 36355 36356 36357 36361 36362 36363 36370 36371 36372 3638 3639 36441 36442 36451 36452 36453 36454 36455 36456 36457 36459 36460 36461 36462 36463 36464 36470 36471 36472 36473 36474 36475 36476 36477 3648 36481 36482 36489 3649 37000 37001 37002 37003 37004 37005 37006 37007 37060 37061 37062 37063 37064 37100 37101 37102 37103 37104 37105 37110 37111 37112 37113 37114 37115 37116 37120 37121 37122 37123 37124 37130 37131 37132 37133 37140 37141 37142 37143 37144 37145 37146 37148 37149 37150 37151 37152 37153 37154 37155 37156 37157 37158 37160 37161 37162 37170 37171 37172 37173 37181 37182 37189 3719 37234 37240 37241 37242 37243 37244 37245 37250 37251 37252 37253 37254 37255 37256 37261 37262 37263 37264 37271 37272 37273 37274 37275 3728 37281 37289 3729 3732 37400 37401 37402 37403 37404 37405 37410 37411 37412 37413 37414 37420 37421 37422 37423 37430 37431 37432 37433 37434 37441 37443 37444 37445 37446 37450 37451 37452 37453 37454 37455 37456 37481 37482 37483 37484 37485 37486 37487 37489 3749 37511 37512 37513 37514 37515 37516 37520 37521 37522 37551 37552 37553 37554 37555 37556 37557 37561 37569 37581 37589 3759 37621 37622 37630 37631 37632 37633 37634 37635 37636 37640 37641 37642 37643 37644 37645 37646 37647 37650 37651 37652 3766 37681 37682 37689 3769 37700 37701 37702 37703 37704 37710 37711 37712 37713 37714 37715 37716 37721 37722 37723 37724 37741 37742 37743 37749 37751 37752 37753 37754 37761 37762 37763 37771 37772 37773 37775 3779 37800 37801 37802 37803 37804 37805 37806 37807 37808 37810 37811 37812 37813 37814 37815 37816 37817 37818 37820 37821 37822 37823 37824 37830 37831 37832 37833 37834 37835 37840 37841 37842 37843 37844 37845 37850 37851 37852 37853 37854 37855 37856 37860 37861 37862 37863 37871 37872 37873 37881 37882 37883 37884 37885 37886 37887 3789 37911 37912 37913 37914 37915 37916 37919 37921 37922 37923 37924 37925 37926 37927 37929 37931 37932 37933 37934 37939 37940 37941 37942 37943 37945 37946 37949 37950 37951 37952 37953 37954 37955 37956 37957 37958 37959 3798 37990 37991 37992 37993 37999 78193 V411 V425 V430 V456 V4561 V4569 V522 V531 V720
		92	Otitis media and re-lated conditions	0552 38100 38101 38102 38103 38104 38105 38106 38110 38119 38120 38129 3813 3814 38150 38151 38152 38160 38161 38162 38163 3817 38181 38189 3819 38200 38201 38202 3821 3822 3823 3824 3829 38300 38301 38302 3831 38320 38321 38322 38330 38331 38332 38333 38381 38389 3839 38420 38421 38422 38423 38424 38425 38481 38482 3849 38500 38501 38502 38503 38509 38510 38511 38512 38513 38519 38521 38522 38523 38524 3870 3871 3872 3878 3879

		93	Conditions associated with dizziness or vertigo	38600 38601 38602 38603 38604 38610 38611 38612 38619 3862 38630 38631 38632 38633 38634 38635 38640 38641 38642 38643 38648 38650 38651 38652 38653 38654 38655 38656 38658 3868 3869 7804
		94	Other ear and sense organ disorders	38000 38001 38002 38003 38010 38011 38012 38013 38014 38015 38016 38021 38022 38023 38030 38031 38032 38039 3804 38050 38051 38052 38053 38081 38089 3809 38400 38401 38409 3841 38530 38531 38532 38533 38535 38582 38583 38589 3859 38800 38801 38802 38810 38811 38812 3882 38830 38831 38832 38840 38841 38842 38843 38844 38845 3885 38860 38861 38869 38870 38871 38872 3888 3889 38900 38901 38902 38903 38904 38905 38906 38908 38910 38911 38912 38913 38914 38915 38916 38917 38918 3892 38920 38921 38922 3897 3898 3899 V412 V413 V4985 V532 V721 V7211 V7212 V7219
		95	Other nervous system disorders	325 32702 32715 32730 32731 32732 32733 32734 32735 32736 32737 32739 32753 33183 3321 33720 33721 33722 33729 3380 33811 33812 33818 33819 33821 33822 33828 33829 3383 3384 3410 3411 3418 3419 34461 347 34700 34701 34710 34711 3480 3482 3483 34830 34831 34839 3484 3485 3488 34881 34882 34889 3489 3492 34981 34982 34989 3499 3501 3502 3508 3509 3510 3511 3518 3519 3520 3521 3522 3523 3524 3525 3526 3529 3530 3531 3532 3533 3534 3535 3536 3538 3539 3540 3541 3542 3543 3544 3545 3548 3549 3550 3551 3552 3553 3554 3555 3556 3557 35571 35579 3558 3559 3560 3561 3562 3563 3564 3568 3569 3570 3571 3572 3573 3574 3576 3577 3578 35781 35782 35789 3579 3580 35800 35801 3581 3582 35830 35831 35839 3588 3589 3590 3591 3592 35921 35922 35923 35924 35929 3593 3594 3595 3596 35971 35979 3598 35981 35989 3599 7810 7811 7812 7813 7817 7818 7820 7843 7845 78451 78452 78459 78460 78461 78469 7920 7930 79400 79401 79402 79409 79410 79411 79412 79413 79414 79415 79416 79417 79419 7961 79951 79952 79953 79954 79955 79959 V124 V1240 V1241 V1242 V1249 V415 V452 V484 V485 V493 V530 V5301 V5302 V5309
7	Cardiovascular diseases	96	Heart valve disorders	3940 3941 3942 3949 3950 3951 3952 3959 3960 3961 3962 3963 3968 3969 3970 3971 3979 4240 4241 4242 4243 42490 42491 42499 7852 7853 V422 V433
		97	Peri-, endo-, and myocarditis, cardiomyopathy	03282 03640 03641 03642 03643 07420 07421 07422 07423 11281 11503 11504 11513 11514 11593 11594 1303 3910 3911 3912 3918 3919 3920 393 3980 39890 39899 4200 42090 42091 42099 4210 4211 4219 4220 42290 42291 42292 42293 42299 4230 4231 4232 4233 4238 4239 4250 4251 42511 42518 4252 4253 4254 4257 4258 4259 4290

		98	Essential hypertension	4011 4019
		99	Hypertension with complications and secondary hypertension	4010 40200 40201 40210 40211 40290 40291 4030 40300 40301 4031 40310 40311 4039 40390 40391 4040 40400 40401 40402 40403 4041 40410 40411 40412 40413 4049 40490 40491 40492 40493 40501 40509 40511 40519 40591 40599 4372
		100	Acute myocardial infarction	4100 41000 41001 41002 4101 41010 41011 41012 4102 41020 41021 41022 4103 41030 41031 41032 4104 41040 41041 41042 4105 41050 41051 41052 4106 41060 41061 41062 4107 41070 41071 41072 4108 41080 41081 41082 4109 41090 41091 41092
		101	Coronary atherosclerosis	4110 4111 4118 41181 41189 412 4130 4131 4139 4140 41400 41401 41406 4142 4143 4144 4148 4149 V4581 V4582
		102	Nonspecific chest pain	78650 78651 78659
		103	Pulmonary heart disease	4150 4151 41512 41513 41519 4160 4161 4162 4168 4169 4170 4171 4178 4179
		104	Other and ill-defined heart disease	41410 41411 41412 41419 4291 4292 4293 4295 4296 42971 42979 42981 42982 42983 42989 4299
		105	Conduction disorders	4260 42610 42611 42612 42613 4262 4263 4264 42650 42651 42652 42653 42654 4266 4267 42681 42682 42689 4269 V450 V4500 V4501 V4502 V4509 V533 V5331 V5332 V5339
		106	Cardiac dysrhythmias	4270 4271 4272 42731 42732 42760 42761 42769 42781 42789 4279 7850 7851

				42741 42742 4275
		107	Cardiac arrest and ventricular fibrillation	
		108	Congestive heart failure	39891 4280 4281 42820 42821 42822 42823 42830 42831 42832 42833 42840 42841 42842 42843 4289
		109	Stroke	4660 34661 34662 34663 430 431 4320 4321 4329 43301 43311 43321 43331 43381 43391 4340 43400 43401 4341 43410 43411 4349 43490 43491 436
		110	Occlusion or stenosis of precerebral arteries	330 43300 4331 43310 4332 43320 4333 43330 4338 43380 4339 43390
		111	Other and ill-defined cerebrovascular disease	4370 4371 4373 4374 4375 4376 4377 4378 4379
		112	Transient cerebral ischemia	4350 4351 4352 4353 4358 4359
		113	Late effects of cerebrovascular disease	438 4380 43810 43811 43812 43813 43814 43819 43820 43821 43822 43830 43831 43832 43840 43841 43842 43850 43851 43852 43853 4386 4387 43881 43882 43883 43884 43885 43889 4389
		114	Atherosclerosis	400 4401 4402 44020 44021 44022 44023 44029 4404 4408 4409 4439 5570 5571 5579

		115	Aortic, peripheral, and visceral artery aneurysms	4410 44100 44101 44102 44103 4411 4412 4413 4414 4415 4416 4417 4419 4420 4421 4422 4423 44281 44282 44283
		116	Aortic and peripheral arterial embolism or thrombosis	4440 44401 44409 4441 44421 44422 44481 44489 4449 44501 44502 44581 44589
		117	Other circulatory disease	4430 4431 44381 44382 44389 4460 4461 4462 44620 44621 44629 4463 4464 4465 4466 4467 4470 4471 4472 4473 4474 4475 4476 4478 4479 4480 4481 4489 4580 4581 4588 4589 4590 45989 4599 7859 79430 79431 79439 7962 V125 V1250 V1253 V1254 V1259 V151 V421 V432 V4321 V4322 V434 V717
		118	Phlebitis, thrombophlebitis and thromboembolism	4510 45111 45119 4512 45181 45182 45183 45184 45189 4519 452 4530 4531 4532 4533 45340 45341 45342 45350 45351 45352 4536 45371 45372 45373 45374 45375 45376 45377 45379 4538 45381 45382 45383 45384 45385 45386 45387 45389 4539 V1251 V1252 V1255
		119	Varicose veins of lower extremity	4540 4541 4542 4548 4549
		120	Hemorrhoids	4550 4551 4552 4553 4554 4555 4556 4557 4558 4559
		121	Other diseases of veins and lymphatics	4563 4564 4565 4566 4568 4570 4571 4572 4578 4579 4591 45910 45911 45912 45913 45919 4592 45930 45931 45932 45933 45939 45981

8	Respiratory diseases	122	Pneumonia	00322 0203 0204 0205 0212 0221 0310 0391 0521 0551 0730 0830 1124 1140 1144 1145 11505 11515 11595 1304 1363 4800 4801 4802 4803 4808 4809 481 4820 4821 4822 4823 48230 48231 48232 48239 4824 48240 48241 48242 48249 4828 48281 48282 48283 48284 48289 4829 483 4830 4831 4838 4841 4843 4845 4846 4847 4848 485 486 5130 5171
		123	Influenza	4870 4871 4878 488 4880 48801 48802 48809 4881 48811 48812 48819 48881 48882 48889
		124	Acute and chronic tonsillitis	463 4740 47400 47401 47402 47410 47411 47412 4742 4748 4749 475
		125	Acute bronchitis	4660 4661 46611 46619
		126	Other upper respiratory infections	0320 0321 0322 0323 0340 460 4610 4611 4612 4613 4618 4619 462 4640 46400 46401 46410 46411 46420 46421 46430 46431 4644 46450 46451 4650 4658 4659 4730 4731 4732 4733 4738 4739 78491
		127	COPD	490 4910 4911 4912 49120 49121 49122 4918 4919 4920 4928 494 4940 4941 496
		128	Asthma	9300 49301 49302 49310 49311 49312 49320 49321 49322 49381 49382 49390 49391 49392
		129	Aspiration pneumonitis (food/vomitus)	5070
		130	Pleurisy, pneumothorax, pulmonary collapse	5100 5109 5110 5111 5118 51189 5119 5120 5128 51281 51282 51283 51284 51289 5180 5181 5182
		131	Respiratory failure	5173 5185 51851 51852 51853 51881 51882 51883 51884 7991 V461 V4611 V4612 V4613 V4614 V462
		132	Lung disease due to external agents	4950 4951 4952 4953 4954 4955 4956 4957 4958 4959 500 501 502 503 504 505 5060 5061 5062 5063 5064 5069 5071 5078 5080 5081 5082 5088 5089

		133	Other lower respiratory disease	5131 514 515 5160 5161 5162 5163 51630 51631 51632 51633 51634 51635 51636 51637 5164 5165 51661 51662 51663 51664 51669 5168 5169 5172 5178 5183 5184 51889 5194 5198 5199 7825 78600 78601 78602 78603 78604 78605 78606 78607 78609 7862 7863 78630 78631 78639 7864 78652 7866 7867 7868 7869 7931 79311 79319 7942 V126 V1260 V1261 V1269 V426
		134	Other upper respiratory disease	470 4710 4711 4718 4719 4720 4721 4722 4760 4761 4770 4772 4778 4779 4780 4781 47811 47819 47820 47821 47822 47824 47825 47826 47829 47830 47831 47832 47833 47834 4784 4785 4786 47870 47871 47874 47875 47879 4788 4789 5191 51911 51919 5192 5193 7841 78440 78441 78442 78443 78444 78449 7847 7848 7849 78499 7861 V414 V440 V550
9	Digestive system diseases	135	Intestinal infection	0010 0011 0019 0020 0021 0022 0023 0029 0030 00320 00329 0038 0039 0040 0041 0042 0043 0048 0049 0050 0051 0052 0053 0054 0058 00581 00589 0059 0060 0061 0062 0063 0064 0065 0066 0068 0069 0070 0071 0072 0073 0074 0075 0078 0079 0080 00800 00801 00802 00803 00804 00809 0081 0082 0083 00841 00842 00843 00844 00845 00846 00847 00849 0085 0086 00861 00862 00863 00864 00865 00866 00867 00869 0088 0090 0091 0092 0093 0211 0222
		136	Disorders of teeth and jaw	5200 5201 5202 5203 5204 5205 5206 5207 5208 5209 5210 52100 52101 52102 52103 52104 52105 52106 52107 52108 52109 5211 52110 52111 52112 52113 52114 52115 5212 52120 52121 52122 52123 52124 52125 5213 52130 52131 52132 52133 52134 52135 5214 52140 52141 52142 52149 5215 5216 5217 5218 52181 52189 5219 5220 5221 5222 5223 5224 5225 5226 5227 5228 5229 5230 52300 52301 5231 52310 52311 5232 52320 52321 52322 52323 52324 52325 5233 52330 52331 52332 52333 5234 52340 52341 52342 5235 5236 5238 5239 5240 52400 52401 52402 52403 52404 52405 52406 52407 52409 5241 52410 52411 52412 52419 5242 52420 52421 52422 52423 52424 52425 52426 52427 52428 52429 5243 52430 52431 52432 52433 52434 52435 52436 52437 52439 5244 5245 52450 52451 52452 52453 52454 52455 52456 52457 52459 5246 52460 52461 52462 52463 52464 52469 52470 52471 52472 52473 52474 52475 52476 52479 5248 52481 52482 52489 5249 5250 5251 52510 52511 52512 52513 52519 5252 52520 52521 52522 52523 52524 52525 52526 5253 52540 52541 52542 52543 52544 52550 52551 52552 52553 52554 52560 52561 52562 52563 52564 52565 52566 52567 52569 52571 52572 52573 52579 5258 5259 5260 5261 5262 5263 5264 5265 52661 52662 52663 52669 52681 52689 5269 78492 V523 V534 V585 V722
		137	Diseases of mouth (excluding dental)	5270 5271 5272 5273 5274 5275 5276 5277 5278 5279 5280 52800 52809 5281 5282 5283 5284 5285 5286 5287 52871 52872 52879 5288 5289 5290 5291 5292 5293 5294 5295 5296 5298 5299 7924
		138	Esophageal disorders	4561 45621 5300 5301 53010 53011 53012 53013 53019 5302 53020 53021 5303 5304 5305 5306 5308 53081 53083 53084 53085 53089 5309

		139	Gastroduodenal ulcer except hemorrhage	53110 53111 53130 53131 53150 53151 53170 53171 53190 53191 53210 53211 53230 53231 53250 53251 53270 53271 53290 53291 53310 53311 53330 53331 53350 53351 53370 53371 53390 53391 53410 53411 53430 53431 53450 53451 53470 53471 53490 53491 V1271
		140	Gastritis and duodenitis	5350 53500 53501 5351 53510 53511 5352 53520 53521 5354 53540 53541 5355 53550 53551 5356 53560 53561 53570 53571
		141	Other disorders of stomach and duodenum	5360 5361 5362 5363 5368 5369 5370 5371 5372 5373 5374 5375 5376 53781 53782 53783 53784 53789 5379
		142	Appendicitis and other appendiceal conditions	5400 5401 5409 541 542 5430 5439
		143	Abdominal hernia	55000 55001 55002 55003 55010 55011 55012 55013 55090 55091 55092 55093 55100 55101 55102 55103 5511 55120 55121 55129 5513 5518 5519 55200 55201 55202 55203 5521 55220 55221 55229 5523 5528 5529 55300 55301 55302 55303 5531 55320 55321 55329 5533 5538 5539
		144	Regional enteritis and ulcerative colitis	5550 5551 5552 5559 556 5560 5561 5562 5563 5564 5565 5566 5568 5569
		145	Bowel obstruction	5600 5601 5602 56030 56031 56032 56039 56081 56089 5609
		146	Diverticulosis and diverticulitis	56200 56201 56202 56203 56210 56211 56212 56213
		147	Anal and rectal conditions	5646 5650 5651 566 5690 5691 5692 56941 56942 56943 56944 56949

		148	Peritonitis and intestinal abscess	03283 5670 5671 5672 56721 56722 56723 56729 56738 56739 5678 56781 56782 56789 5679 5695
		149	Biliary tract disease	57400 57401 57410 57411 57420 57421 57430 57431 57440 57441 57450 57451 57460 57461 57470 57471 57480 57481 57490 57491 5750 5751 57510 57511 57512 5752 5753 5754 5755 5756 5758 5759 5760 5761 5762 5763 5764 5765 5768 5769 7933
		151	Other liver diseases	570 5715 5716 5718 5719 5720 5721 5722 5723 5724 5728 5730 5734 5735 5738 5739 7824 7891 7895 78959 7904 7905 7948 V427
		152	Pancreatic disorders not diabetes	5770 5771 5772 5778 5779 5794
		153	Gastrointestinal hemorrhage	4560 45620 5307 53082 53100 53101 53120 53121 53140 53141 53160 53161 53200 53201 53220 53221 53240 53241 53260 53261 53300 53301 53320 53321 53340 53341 53360 53361 53400 53401 53420 53421 53440 53441 53460 53461 5693 5780 5781 5789
		154	Noninfectious gastroenteritis	55841 55842 5589
		155	Other gastrointestinal disorders	538 5581 5582 5640 56400 56401 56402 56409 5641 5645 5647 5648 56481 56489 5649 5680 56881 56882 56889 5689 56981 56982 56983 56984 56985 56986 56987 56989 5699 5790 5791 5792 5798 5799 7871 7872 78720 78721 78722 78723 78724 78729 7873 7874 7875 7876 78760 78761 78762 78763 7877 7879 78791 78799 7892 7893 78930 78931 78932 78933 78934 78935 78936 78937 78939 7894 78940 78941 78942 78943 78944 78945 78946 78947 78949 7899 7921 7934 7936 V127 V1270 V1279 V416 V441 V442 V443 V444 V453 V473 V535 V5350 V5351 V5359 V551 V552 V553 V554
10	Genitourinary diseases	156	Nephritis, nephrosis, renal sclerosis	5800 5804 58081 58089 5809 5810 5811 5812 5813 58181 58189 5819 5820 5821 5822 5824 58281 58289 5829 5830 5831 5832 5834 5836 5837 58381 58389 5839 587

		157	Renal failure (acute/unspecified)	5845 5846 5847 5848 5849 586
		158	Chronic kidney disease	585 5851 5852 5853 5854 5855 5856 5859 7925 V420 V451 V4511 V4512 V560 V561 V562 V5631 V5632 V568
		159	Urinary tract infections	03284 59000 59001 59010 59011 5902 5903 59080 59081 5909 5950 5951 5952 5953 5954 59581 59582 59589 5959 5970 59780 59781 59789 59800 59801 5990
		160	Calculus of urinary tract	5920 5921 5929 5940 5941 5942 5948 5949 7880 V1301
		161	Other diseases of kidney and ureters	5880 5881 5888 58881 58889 5889 5890 5891 5899 591 5930 5931 5932 5933 5934 5935 5936 5937 59370 59371 59372 59373 59381 59382 59389 5939
		162	Other diseases of bladder and urethra	5960 5961 5962 5963 5964 5965 59651 59652 59653 59654 59655 59659 5966 5967 5968 59689 5969 5981 5982 5988 5989 5991 5992 5993 5994 5995 59981 59982 59983 59984
		163	Genitourinary symptoms and ill-defined conditions	5996 59960 59969 5997 59970 59971 59972 5998 59989 5999 7881 7882 78820 78821 78829 7883 78830 78831 78832 78833 78834 78835 78836 78837 78838 78839 7884 78841 78842 78843 7885 7886 78861 78862 78863 78864 78865 78869 7887 7888 7889 78891 78899 7910 7911 7912 7913 7914 7917 7919 7935 7944 V130 V1300 V1302 V1303 V1309 V417 V435 V445 V4450 V4451 V4452 V4459 V446 V474 V475 V536 V555 V556
		164	Hyperplasia of prostate	600 6000 60000 60001 6001 60010 60011 6002 60020 60021 6003 6009 60090 60091

		165	Inflammatory conditions of male genital organs	6010 6011 6012 6013 6014 6018 6019 6031 6040 60490 60491 60499 6071 6072 6080 6084
		166	Other male genital disorders	6020 6021 6022 6023 6028 6029 6030 6038 6039 605 6060 6061 6068 6069 6070 6073 60781 60782 60783 60784 60785 60789 6079 6081 6082 60820 60821 60822 60823 60824 6083 60881 60882 60883 60884 60885 60886 60887 60889 6089 7922
		167	Nonmalignant breast conditions	6100 6101 6102 6103 6104 6108 6109 6110 6111 6112 6113 6114 6115 6116 61171 61172 61179 6118 61181 61182 61183 61189 6119 6120 6121 7938 79380 79381 79382 79389
		168	Inflammatory diseases of female pelvic organs	6140 6141 6142 6143 6144 6145 6146 6147 6148 6149 6150 6151 6159 6160 61610 61611 6162 6163 6164 61650 61651 6168 61681 61689 6169 62571
		169	Endometriosis	6170 6171 6172 6173 6174 6175 6176 6178 6179
		170	Prolapse of female genital organs	6180 61800 61801 61802 61803 61804 61805 61809 6181 6182 6183 6184 6185 6186 6187 6188 61881 61882 61883 61884 61889 6189
		171	Menstrual disorders	6253 6260 6261 6262 6263 6264 6265 6266 6268 6269
		172	Ovarian cyst	6200 6201 6202
		173	Menopausal disorders	25631 25639 6270 6271 6272 6273 6274 6278 6279 V074
		174	Female infertility	6280 6281 6282 6283 6284 6288 6289

		175	Other female genital disorders	6190 6191 6192 6198 6199 6203 6204 6205 6206 6207 6208 6209 6210 6211 6212 6213 62130 62131 62132 62133 62134 62135 6214 6215 6216 6217 6218 6219 6220 6221 62210 62211 62212 6222 6223 6224 6225 6226 6227 6228 6229 6230 6231 6232 6233 6234 6235 6236 6237 6238 6239 6240 62401 62402 62409 6241 6242 6243 6244 6245 6246 6248 6249 6250 6251 6252 6254 6255 6256 62570 62579 6258 6259 6267 6290 6291 62920 62921 62922 62923 62929 6298 62981 62989 6299 79500 79501 79502 79503 79504 79507 79508 79509 V132 V1321 V1322 V1323 V1324 V1329 V557 V723
12	Skin and subcu-taneous tissue diseases	197	Skin infections	0201 0210 0220 0311 03285 035 0390 6800 6801 6802 6803 6804 6805 6806 6807 6808 6809 68100 68101 68102 68110 68111 6819 6820 6821 6822 6823 6824 6825 6826 6827 6828 6829 684 6850 6851 6860 68600 68601 68609 6861 6868 6869
		198	Other inflammatory condition of skin	690 69010 69011 69012 69018 6908 69276 69277 6940 6941 6942 6943 6944 6945 69460 69461 6948 6949 6950 6951 69510 69511 69512 69513 69514 69515 69519 6952 6953 6954 69550 69551 69552 69553 69554 69555 69556 69557 69558 69559 69581 69589 6959 6960 6961 6962 6963 6964 6965 6968 6970 6971 6978 6979 6980 6981 6982 6983 6984 6988 6989
		199	Chronic ulcer of skin	7070 70700 70701 70702 70703 70704 70705 70706 70707 70709 7071 70710 70711 70712 70713 70714 70715 70719 70720 70721 70722 70723 70724 70725 7078 7079
		200	Other skin disorders	69275 700 7010 7011 7012 7013 7014 7015 7018 7019 702 7020 7021 70211 70219 7028 7030 7038 7039 70400 70401 70402 70409 7041 7042 7043 70441 70442 7048 7049 7050 7051 70521 70522 70581 70582 70583 70589 7059 7060 7061 7062 7063 7068 7069 7090 70900 70901 70909 7091 7092 7093 7094 7098 7099 7808 7821 7822 V133 V423
13	Musculoskeletal system and con-nective tissue diseases	201	Infective arthritis and osteomyelitis	0323 00324 0261 03682 05671 71100 71101 71102 71103 71104 71105 71106 71107 71108 71109 71110 71111 71112 71113 71114 71115 71116 71117 71118 71119 71120 71121 71122 71123 71124 71125 71126 71127 71128 71129 71130 71131 71132 71133 71134 71135 71136 71137 71138 71139 71140 71141 71142 71143 71144 71145 71146 71147 71148 71149 71150 71151 71152 71153 71154 71155 71156 71157 71158 71159 71160 71161 71162 71163 71164 71165 71166 71167 71168 71169 71170 71171 71172 71173 71174 71175 71176 71177 71178 71179 71180 71181 71182 71183 71184 71185 71186 71187 71188 71189 71190 71191 71192 71193 71194 71195 71196 71197 71198 71199 73000 73001 73002 73003 73004 73005 73006 73007 73008 73009 73010 73011 73012 73013 73014 73015 73016 73017 73018 73019 73020 73021 73022 73023 73024 73025 73026 73027 73028 73029 73030 73031 73032 73033 73034 73035 73036 73037 73038 73039 73070 73071 73072 73073 73074 73075 73076 73077 73078 73079 73080 73081 73082 73083 73084 73085 73086 73087 73088 73089 73090 73091 73092 73093 73094 73095 73096 73097 73098 73099

		202	Rheumatoid arthritis and related disease	7140 7141 7142 71430 71431 71432 71433 7144 71481 71489 7149 7200
		203	Osteoarthritis	71500 71504 71509 71510 71511 71512 71513 71514 71515 71516 71517 71518 71520 71521 71522 71523 71524 71525 71526 71527 71528 71530 71531 71532 71533 71534 71535 71536 71537 71538 71580 71589 71590 71591 71592 71593 71594 71595 71596 71597 71598 V134
		204	Other non-traumatic joint disorders	7130 7131 7132 7133 7134 7135 7136 7137 7138 71600 71601 71602 71603 71604 71605 71606 71607 71608 71609 71620 71621 71622 71623 71624 71625 71626 71627 71628 71629 71630 71631 71632 71633 71634 71635 71636 71637 71638 71639 71640 71641 71642 71643 71644 71645 71646 71647 71648 71649 71650 71651 71652 71653 71654 71655 71656 71657 71658 71659 71660 71661 71662 71663 71664 71665 71666 71667 71668 71680 71681 71682 71683 71684 71685 71686 71687 71688 71689 71690 71691 71692 71693 71694 71695 71696 71697 71698 71699 71810 71811 71812 71813 71814 71815 71817 71818 71819 71820 71821 71822 71823 71824 71825 71826 71827 71828 71829 71850 71851 71852 71853 71854 71855 71856 71857 71858 71859 71860 71865 71870 71871 71872 71873 71874 71875 71876 71877 71878 71879 71880 71881 71882 71883 71884 71885 71886 71887 71888 71889 71890 71891 71892 71893 71894 71895 71897 71898 71899 71900 71901 71902 71903 71904 71905 71906 71907 71908 71909 71910 71911 71912 71913 71914 71915 71916 71917 71918 71919 71920 71921 71922 71923 71924 71925 71926 71927 71928 71929 71930 71931 71932 71933 71934 71935 71936 71937 71938 71939 71940 71941 71942 71943 71944 71945 71946 71947 71948 71949 71950 71951 71952 71953 71954 71955 71956 71957 71958 71959 71960 71961 71962 71963 71964 71965 71966 71967 71968 71969 7197 71970 71975 71976 71977 71978 71979 71980 71981 71982 71983 71984 71985 71986 71987 71988 71989 71990 71991 71992 71993 71994 71995 71996 71997 71998 71999
		205	Back problems	7201 7202 72081 72089 7209 7210 7211 7212 7213 72141 72142 7215 7216 7217 7218 72190 72191 7220 72210 72211 7222 72230 72231 72232 72239 7224 72251 72252 7226 72270 72271 72272 72273 72280 72281 72282 72283 72290 72291 72292 72293 7230 7231 7232 7233 7234 7235 7236 7237 7238 7239 72400 72401 72402 72403 72409 7241 7242 7243 7244 7245 7246 72470 72471 72479 7248 7249
		206	Osteoporosis	73300 73301 73302 73303 73309
		207	Pathological fracture	7331 73310 73311 73312 73313 73314 73315 73316 73319 73393 73394 73395 73396 73397 73398 V1351 V1352

		208	Acquired foot deformities	7271 734 7350 7351 7352 7353 7354 7355 7358 7359 73670 73671 73672 73673 73674 73675 73676 73679
		209	Other acquired deformities	71840 71841 71842 71843 71844 71845 71846 71847 71848 71849 73600 73601 73602 73603 73604 73605 73606 73607 73609 7361 73620 73621 73622 73629 73630 73631 73632 73639 73641 73642 7365 7366 73681 73689 7369 7370 73710 73711 73712 73719 73720 73721 73722 73729 73733 73734 73739 73740 73741 73742 73743 7378 7379 7380 7381 73810 73811 73812 73819 7382 7383 7384 7385 7386 7387 7388 7389
		210	Systemic lupus erythematosus and connective tissue disorders	7100 7101 7102 7103 7104 7108 7109
		211	Other connective tissue disease	32752 56731 7105 725 7260 72610 72611 72612 72613 72619 7262 72630 72631 72632 72633 72639 7264 7265 72660 72661 72662 72663 72664 72665 72669 72670 72671 72672 72673 72679 7268 72690 72691 72700 72701 72702 72703 72704 72705 72706 72709 7272 7273 72740 72741 72742 72743 72749 72750 72751 72759 72760 72761 72762 72763 72764 72765 72766 72767 72768 72769 72781 72782 72783 72789 7279 7280 72810 72811 72812 72813 72819 7282 7283 7284 7285 7286 72871 72879 72881 72882 72883 72884 72885 72886 72887 72888 72889 7289 7290 7291 7292 72930 72931 72939 7294 7295 7296 72971 72972 72973 72979 72981 72982 72989 7299 72990 72991 72992 72999 7819 78191 78192 78194 78199 7937 V135 V1359 V436 V4360 V4361 V4362 V4363 V4364 V4365 V4366 V4369 V437 V454 V481 V482 V483 V490 V491 V492 V495 V4960 V4961 V4962 V4963 V4964 V4965 V4966 V4967 V4970 V4971 V4972 V4973 V4974 V4975 V4976 V4977 V537
		212	Other bone disease and musculoskeletal deformities	7310 7311 7312 7313 7318 7320 7321 7322 7323 7324 7325 7326 7327 7328 7329 73320 73321 73322 73329 7333 73340 73341 73342 73343 73344 73345 73349 7335 7336 7337 73381 73382 73390 73391 73392 73399 73730 73731 73732 7390 7391 7392 7393 7394 7395 7396 7397 7398 7399 V424 V486 V487 V494 V8821 V8822 V8829

		225	Joint disorders and dislocations	71610 71611 71612 71613 71614 71615 71616 71617 71618 71619 7170 7171 7172 7173 71740 71741 71742 71743 71749 7175 7176 7177 71781 71782 71783 71784 71785 71789 7179 71800 71801 71802 71803 71804 71805 71807 71808 71809 71830 71831 71832 71833 71834 71835 71836 71837 71838 71839 8300 8301 83100 83101 83102 83103 83104 83109 83110 83111 83112 83113 83114 83119 83200 83201 83202 83203 83204 83209 83210 83211 83212 83213 83214 83219 8322 83300 83301 83302 83303 83304 83305 83309 83310 83311 83312 83313 83314 83315 83319 83400 83401 83402 83410 83411 83412 83500 83501 83502 83503 83510 83511 83512 83513 8360 8361 8362 8363 8364 83650 83651 83652 83653 83654 83659 83660 83661 83662 83663 83664 83669 8370 8371 83800 83801 83802 83803 83804 83805 83806 83809 83810 83811 83812 83813 83814 83815 83816 83819 83900 83901 83902 83903 83904 83905 83906 83907 83908 83910 83911 83912 83913 83914 83915 83916 83917 83918 83920 83921 83930 83931 83940 83941 83942 83949 83950 83951 83952 83959 83961 83969 83971 83979 8398 8399 9056
16	Injuries	226	Fracture of neck of femur hip	82000 82001 82002 82003 82009 82010 82011 82012 82013 82019 82020 82021 82022 82030 82031 82032 8208 8209 9053 V5413 V5423
		227	Spinal cord injury	34939 80600 80601 80602 80603 80604 80605 80606 80607 80608 80609 80610 80611 80612 80613 80614 80615 80616 80617 80618 80619 80620 80621 80622 80623 80624 80625 80626 80627 80628 80629 80630 80631 80632 80633 80634 80635 80636 80637 80638 80639 8064 8065 80660 80661 80662 80669 80670 80671 80672 80679 8068 8069 9072 95200 95201 95202 95203 95204 95205 95206 95207 95208 95209 95210 95211 95212 95213 95214 95215 95216 95217 95218 95219 9522 9523 9524 9528 9529
		228	Skull and face fractures	80000 80001 80002 80003 80004 80005 80006 80009 80050 80051 80052 80053 80054 80055 80056 80059 80100 80101 80102 80103 80104 80105 80106 80109 80150 80151 80152 80153 80154 80155 80156 80159 8020 8021 80220 80221 80222 80223 80224 80225 80226 80227 80228 80229 80230 80231 80232 80233 80234 80235 80236 80237 80238 80239 8024 8025 8026 8027 8028 8029 80300 80301 80302 80303 80304 80305 80306 80309 80350 80351 80352 80353 80354 80355 80356 80359 80400 80401 80402 80403 80404 80405 80406 80409 80450 80451 80452 80453 80454 80455 80456 80459 9050

		229	Fracture of upper limb	81000 81001 81002 81003 81010 81011 81012 81013 81100 81101 81102 81103 81109 81110 81111 81112 81113 81119 81200 81201 81202 81203 81209 81210 81211 81212 81213 81219 81220 81221 81230 81231 81240 81241 81242 81243 81244 81249 81250 81251 81252 81253 81254 81259 81300 81301 81302 81303 81304 81305 81306 81307 81308 81310 81311 81312 81313 81314 81315 81316 81317 81318 81320 81321 81322 81323 81330 81331 81332 81333 81340 81341 81342 81343 81344 81345 81346 81347 81350 81351 81352 81353 81354 81380 81381 81382 81383 81390 81391 81392 81393 81400 81401 81402 81403 81404 81405 81406 81407 81408 81409 81410 81411 81412 81413 81414 81415 81416 81417 81418 81419 81500 81501 81502 81503 81504 81509 81510 81511 81512 81513 81514 81519 81600 81601 81602 81603 81610 81611 81612 81613 8170 8171 8180 8181 8190 8191 9052 V5410 V5411 V5412 V5420 V5421 V5422
		230	Fracture of lower limb	82100 82101 82110 82111 82120 82121 82122 82123 82129 82130 82131 82132 82133 82139 8220 8221 82300 82301 82302 82310 82311 82312 82320 82321 82322 82330 82331 82332 82340 82341 82342 82380 82381 82382 82390 82391 82392 8240 8241 8242 8243 8244 8245 8246 8247 8248 8249 8250 8251 82520 82521 82522 82523 82524 82525 82529 82530 82531 82532 82533 82534 82535 82539 8260 8261 8270 8271 9054 V5414 V5415 V5416 V5424 V5425 V5426
		231	Other fractures	80500 80501 80502 80503 80504 80505 80506 80507 80508 80510 80511 80512 80513 80514 80515 80516 80517 80518 8052 8053 8054 8055 8056 8057 8058 8059 80700 80701 80702 80703 80704 80705 80706 80707 80708 80709 80710 80711 80712 80713 80714 80715 80716 80717 80718 80719 8072 8073 8074 8075 8076 8080 8081 8082 8083 80841 80842 80843 80844 80849 80851 80852 80853 80854 80859 8088 8089 8090 8091 8280 8281 8290 8291 9051 9055 V540 V5401 V5402 V5409 V5417 V5419 V5427 V5429 V664 V674
		232	Sprains and strains	8400 8401 8402 8403 8404 8405 8406 8407 8408 8409 8410 8411 8412 8413 8418 8419 84200 84201 84202 84209 84210 84211 84212 84213 84219 8430 8431 8438 8439 8440 8441 8442 8443 8448 8449 84500 84501 84502 84503 84509 84510 84511 84512 84513 84519 8460 8461 8462 8463 8468 8469 8470 8471 8472 8473 8474 8479 8480 8481 8482 8483 84840 84841 84842 84849 8485 8488 8489 9057

		233	Intracranial injury	80010 80011 80012 80013 80014 80015 80016 80019 80020 80021 80022 80023 80024 80025 80026 80029 80030 80031 80032 80033 80034 80035 80036 80039 80040 80041 80042 80043 80044 80045 80046 80049 80060 80061 80062 80063 80064 80065 80066 80069 80070 80071 80072 80073 80074 80075 80076 80079 80080 80081 80082 80083 80084 80085 80086 80089 80090 80091 80092 80093 80094 80095 80096 80099 80110 80111 80112 80113 80114 80115 80116 80119 80120 80121 80122 80123 80124 80125 80126 80129 80130 80131 80132 80133 80134 80135 80136 80139 80140 80141 80142 80143 80144 80145 80146 80149 80160 80161 80162 80163 80164 80165 80166 80169 80170 80171 80172 80173 80174 80175 80176 80179 80180 80181 80182 80183 80184 80185 80186 80189 80190 80191 80192 80193 80194 80195 80196 80199 80310 80311 80312 80313 80314 80315 80316 80319 80320 80321 80322 80323 80324 80325 80326 80329 80330 80331 80332 80333 80334 80335 80336 80339 80340 80341 80342 80343 80344 80345 80346 80349 80360 80361 80362 80363 80364 80365 80366 80369 80370 80371 80372 80373 80374 80375 80376 80379 80380 80381 80382 80383 80384 80385 80386 80389 80390 80391 80392 80393 80394 80395 80396 80399 80410 80411 80412 80413 80414 80415 80416 80419 80420 80421 80422 80423 80424 80425 80426 80429 80430 80431 80432 80433 80434 80435 80436 80439 80440 80441 80442 80443 80444 80445 80446 80449 80460 80461 80462 80463 80464 80465 80466 80469 80470 80471 80472 80473 80474 80475 80476 80479 80480 80481 80482 80483 80484 80485 80486 80489 80490 80491 80492 80493 80494 80495 80496 80499 8500 8501 85011 85012 8502 8503 8504 8505 8509 85100 85101 85102 85103 85104 85105 85106 85109 85110 85111 85112 85113 85114 85115 85116 85119 85120 85121 85122 85123 85124 85125 85126 85129 85130 85131 85132 85133 85134 85135 85136 85139 85140 85141 85142 85143 85144 85145 85146 85149 85150 85151 85152 85153 85154 85155 85156 85159 85160 85161 85162 85163 85164 85165 85166 85169 85170 85171 85172 85173 85174 85175 85176 85179 85180 85181 85182 85183 85184 85185 85186 85189 85190 85191 85192 85193 85194 85195 85196 85199 85200 85201 85202 85203 85204 85205 85206 85209 85210 85211 85212 85213 85214 85215 85216 85219 85220 85221 85222 85223 85224 85225 85226 85229 85230 85231 85232 85233 85234 85235 85236 85239 85240 85241 85242 85243 85244 85245 85246 85249 85250 85251 85252 85253 85254 85255 85256 85259 85300 85301 85302 85303 85304 85305 85306 85309 85310 85311 85312 85313 85314 85315 85316 85319 85400 85401 85402 85403 85404 85405 85406 85409 85410 85411 85412 85413 85414 85415 85416 85419 9070 V1552

		234	Crushing injury or internal injury	8600 8601 8602 8603 8604 8605 86100 86101 86102 86103 86110 86111 86112 86113 86120 86121 86122 86130 86131 86132 8620 8621 86221 86222 86229 86231 86232 86239 8628 8629 8630 8631 86320 86321 86329 86330 86331 86339 86340 86341 86342 86343 86344 86345 86346 86349 86350 86351 86352 86353 86354 86355 86356 86359 86380 86381 86382 86383 86384 86385 86389 86390 86391 86392 86393 86394 86395 86399 86400 86401 86402 86403 86404 86405 86409 86410 86411 86412 86413 86414 86415 86419 86500 86501 86502 86503 86504 86509 86510 86511 86512 86513 86514 86519 86600 86601 86602 86603 86610 86611 86612 86613 8670 8671 8672 8673 8674 8675 8676 8677 8678 8679 86800 86801 86802 86803 86804 86809 86810 86811 86812 86813 86814 86819 8690 8691 90000 90001 90002 90003 9001 90081 90082 90089 9009 9010 9011 9012 9013 90140 90141 90142 90181 90182 90183 90189 9019 9020 90210 90211 90219 90220 90221 90222 90223 90224 90225 90226 90227 90229 90231 90232 90233 90234 90239 90240 90241 90242 90249 90250 90251 90252 90253 90254 90255 90256 90259 90281 90282 90287 90289 9029 90300 90301 90302 9031 9032 9033 9034 9035 9038 9039 9040 9041 9042 9043 90440 90441 90442 90450 90451 90452 90453 90454 9046 9047 9048 9049 9064 9080 9081 9082 9083 9084 925 9251 9252 9260 92611 92612 92619 9268 9269 92700 92701 92702 92703 92709 92710 92711 92720 92721 9273 9278 9279 92800 92801 92810 92811 92820 92821 9283 9288 9289 9290 9299
		235	Open wounds of head, neck, and trunk	8700 8701 8702 8703 8704 8708 8709 8710 8711 8712 8713 8714 8715 8716 8717 8719 87200 87201 87202 87210 87211 87212 87261 87262 87263 87264 87269 87271 87272 87273 87274 87279 8728 8729 8730 8731 87320 87321 87322 87323 87329 87330 87331 87332 87333 87339 87340 87341 87342 87343 87344 87349 87350 87351 87352 87353 87354 87359 87360 87361 87362 87363 87364 87365 87369 87370 87371 87372 87373 87374 87375 87379 8738 8739 87400 87401 87402 87410 87411 87412 8742 8743 8744 8745 8748 8749 8750 8751 8760 8761 8770 8771 8780 8781 8782 8783 8784 8785 8786 8787 8788 8789 8790 8791 8792 8793 8794 8795 8796 8797 8798 8799 9060
		236	Open wounds of extremities	88000 88001 88002 88003 88009 88010 88011 88012 88013 88019 88020 88021 88022 88023 88029 88100 88101 88102 88110 88111 88112 88120 88121 88122 8820 8821 8822 8830 8831 8832 8840 8841 8842 8850 8851 8860 8861 8870 8871 8872 8873 8874 8875 8876 8877 8900 8901 8902 8910 8911 8912 8920 8921 8922 8930 8931 8932 8940 8941 8942 8950 8951 8960 8961 8962 8963 8970 8971 8972 8973 8974 8975 8976 8977 9058 9059 9061
		237	Complications (device/implant/graft)	27950 27951 27952 27953 41402 41403 41404 41405 41407 44030 44031 44032 56960 56961 56969 59682 59683 62931 62932 99600 99601 99602 99603 99604 99609 9961 9962 99630 99631 99632 99639 9964 99640 99641 99642 99643 99644 99645 99646 99647 99649 99651 99652 99653 99654 99655 99656 99657 99659 9966 99660 99661 99662 99663 99664 99665 99666 99667 99668 99669 9967 99670 99671 99672 99673 99674 99675 99676 99677 99678 99679 99680 99681 99682 99683 99684 99685 99686 99687 99688 99689 99690 99691 99692 99693 99694 99695 99696 99699 99931 99932 99933

		238	Complications (surgical/medical)	27661 27783 27788 2853 28741 3490 3491 34931 41511 4294 4582 45821 45829 5121 5122 5187 5190 51900 51901 51902 51909 53086 53087 53640 53641 53642 53649 53901 53909 53981 53989 5642 5643 5644 5696 56962 56971 56979 5793 59681 78062 78063 78066 9093 99524 9954 99586 9970 99700 99701 99702 99709 9971 9972 9973 99731 99732 99739 9974 99741 99749 9975 99760 99761 99762 99769 99771 99772 99779 9979 99791 99799 9980 99800 99801 99802 99809 9981 99811 99812 99813 9982 9983 99830 99831 99832 99833 9984 9985 99851 99859 9986 9987 9988 99881 99882 99883 99889 9989 9990 9991 9992 9993 99934 99939 9994 99941 99942 99949 9995 99951 99952 99959 9996 99960 99961 99962 99963 99969 9997 99970 99971 99972 99973 99974 99975 99976 99977 99978 99979 9998 99980 99981 99982 99983 99984 99985 99988 99989 9999 V1553 V1580 V1583 V9001 V9009
		239	Superficial injury, contusion	9062 9063 9100 9101 9102 9103 9104 9105 9106 9107 9108 9109 9110 9111 9112 9113 9114 9115 9116 9117 9118 9119 9120 9121 9122 9123 9124 9125 9126 9127 9128 9129 9130 9131 9132 9133 9134 9135 9136 9137 9138 9139 9140 9141 9142 9143 9144 9145 9146 9147 9148 9149 9150 9151 9152 9153 9154 9155 9156 9157 9158 9159 9160 9161 9162 9163 9164 9165 9166 9167 9168 9169 9170 9171 9172 9173 9174 9175 9176 9177 9178 9179 9180 9181 9182 9189 9190 9191 9192 9193 9194 9195 9196 9197 9198 9199 920 9210 9211 9212 9213 9219 9220 9221 9222 9223 92231 92232 92233 9224 9228 9229 92300 92301 92302 92303 92309 92310 92311 92320 92321 9233 9238 9239 92400 92401 92410 92411 92420 92421 9243 9244 9245 9248 9249

		240	Burns	9065 9066 9067 9068 9069 9400 9401 9402 9403 9404 9405 9409 94100 94101 94102 94103 94104 94105 94106 94107 94108 94109 94110 94111 94112 94113 94114 94115 94116 94117 94118 94119 94120 94121 94122 94123 94124 94125 94126 94127 94128 94129 94130 94131 94132 94133 94134 94135 94136 94137 94138 94139 94140 94141 94142 94143 94144 94145 94146 94147 94148 94149 94150 94151 94152 94153 94154 94155 94156 94157 94158 94159 94200 94201 94202 94203 94204 94205 94209 94210 94211 94212 94213 94214 94215 94219 94220 94221 94222 94223 94224 94225 94229 94230 94231 94232 94233 94234 94235 94239 94240 94241 94242 94243 94244 94245 94249 94250 94251 94252 94253 94254 94255 94259 94300 94301 94302 94303 94304 94305 94306 94309 94310 94311 94312 94313 94314 94315 94316 94319 94320 94321 94322 94323 94324 94325 94326 94329 94330 94331 94332 94333 94334 94335 94336 94339 94340 94341 94342 94343 94344 94345 94346 94349 94350 94351 94352 94353 94354 94355 94356 94359 94400 94401 94402 94403 94404 94405 94406 94407 94408 94410 94411 94412 94413 94414 94415 94416 94417 94418 94420 94421 94422 94423 94424 94425 94426 94427 94428 94430 94431 94432 94433 94434 94435 94436 94437 94438 94440 94441 94442 94443 94444 94445 94446 94447 94448 94450 94451 94452 94453 94454 94455 94456 94457 94458 94500 94501 94502 94503 94504 94505 94506 94509 94510 94511 94512 94513 94514 94515 94516 94519 94520 94521 94522 94523 94524 94525 94526 94529 94530 94531 94532 94533 94534 94535 94536 94539 94540 94541 94542 94543 94544 94545 94546 94549 94550 94551 94552 94553 94554 94555 94556 94559 9460 9461 9462 9463 9464 9465 9470 9471 9472 9473 9474 9478 9479 94800 94810 94811 94820 94821 94822 94830 94831 94832 94833 94840 94841 94842 94843 94844 94850 94851 94852 94853 94854 94855 94860 94861 94862 94863 94864 94865 94866 94870 94871 94872 94873 94874 94875 94876 94877 94880 94881 94882 94883 94884 94885 94886 94887 94888 94890 94891 94892 94893 94894 94895 94896 94897 94898 94899 9490 9491 9492 9493 9494 9495
		241	Poisoning by psycho-tropic agents	9690 96900 96901 96902 96903 96904 96905 96909 9691 9692 9693 9694 9695 9696 9697 96970 96971 96972 96973 96979 9698 9699

		242	Poisoning by other medications and drugs	52801 52802 9090 9095 9600 9601 9602 9603 9604 9605 9606 9607 9608 9609 9610 9611 9612 9613 9614 9615 9616 9617 9618 9619 9620 9621 9622 9623 9624 9625 9626 9627 9628 9629 9630 9631 9632 9633 9634 9635 9638 9639 9640 9641 9642 9643 9644 9645 9646 9647 9648 9649 9651 9654 9655 9656 96561 96569 9657 9658 9659 9660 9661 9662 9663 9664 9670 9671 9672 9673 9674 9675 9676 9678 9679 9680 9681 9682 9683 9684 9685 9686 9687 9689 9700 9701 9708 97081 97089 9709 9710 9711 9712 9713 9719 9720 9721 9722 9723 9724 9725 9726 9727 9728 9729 9730 9731 9732 9733 9734 9735 9736 9738 9739 9740 9741 9742 9743 9744 9745 9746 9747 9750 9751 9752 9753 9754 9755 9756 9757 9758 9760 9761 9762 9763 9764 9765 9766 9767 9768 9769 9770 9771 9772 9773 9774 9778 9779 9780 9781 9782 9783 9784 9785 9786 9788 9789 9790 9791 9792 9793 9794 9795 9796 9797 9799 9952 99520 99521 99522 99523 99527 99529
		243	Poisoning by nonmedicinal substances	9091 9801 9802 9803 9808 9809 981 9820 9821 9822 9823 9824 9828 9830 9831 9832 9839 9840 9841 9848 9849 9850 9851 9852 9853 9854 9855 9856 9858 9859 986 9870 9871 9872 9873 9874 9875 9876 9877 9878 9879 9880 9881 9882 9888 9889 9890 9891 9892 9893 9894 9895 9896 9897 9898 98981 98982 98983 98984 98989 9899
		244	Other injuries and conditions due to external causes	7960 7990 79901 79902 9071 9073 9074 9075 9079 9085 9086 9089 9092 9094 9099 9300 9301 9302 9308 9309 931 932 9330 9331 9340 9341 9348 9349 9350 9351 9352 936 937 938 9390 9391 9392 9393 9399 9500 9501 9502 9503 9509 9510 9511 9512 9513 9514 9515 9516 9517 9518 9519 9530 9531 9532 9533 9534 9535 9538 9539 9540 9541 9548 9549 9550 9551 9552 9553 9554 9555 9556 9557 9558 9559 9560 9561 9562 9563 9564 9565 9568 9569 9570 9571 9578 9579 9580 9581 9582 9583 9584 9585 9586 9587 9588 95890 95891 95892 95893 95899 9590 95901 95909 9591 95911 95912 95913 95914 95919 9592 9593 9594 9595 9596 9597 9598 9599 990 9910 9911 9912 9913 9914 9915 9916 9918 9919 9920 9921 9922 9923 9924 9925 9926 9927 9928 9929 9930 9931 9932 9933 9934 9938 9939 9940 9941 9942 9943 9944 9945 9946 9947 9948 9949 9955 99550 99551 99552 99553 99554 99555 99559 99580 99581 99582 99583 99584 99585 99589 99590 99593 99594 V155 V1551 V1559 V156 V1588 V713 V714 V715 V716 V9010 V9011 V9012 V902 V9031 V9032 V9033 V9039 V9081 V9083 V9089 V909
